# Screening of *Streptococcus Suis* serotype 2 resistance genes with GWAS and transcriptomic microarray analysis

**DOI:** 10.1186/s12864-018-5339-9

**Published:** 2018-12-12

**Authors:** Zhe Ma, Haodan Zhu, Yiqi Su, Yu Meng, Huixing Lin, Kongwang He, Hongjie Fan

**Affiliations:** 1 MOE Joint International Research Laboratory of Animal Health and Food Safety, College of Veterinary Medicine, Nanjing Agricultural University, Nanjing, 210095 China; 2 Ministry of Agriculture Key Laboratory of Animal Bacteriology, Nanjing, 210095 China; 3 Jiangsu Co-innovation Center for Prevention and Control of Important Animal Infectious Diseases and Zoonoses, Yangzhou, 225009 China; 4 Jiangsu Academy Agricultural Sciences, Nanjing, 210095 China

**Keywords:** *Streptococcus suis* serotype 2, Resistance genes, Genome-wide association study, Transcriptome analysis

## Abstract

**Background:**

Swine streptococcosis has caused great economic loss in the swine industry, and the major pathogen responsible for this disease is *Streptococcus Suis* serotype 2 (SS2). Disease resistance breeding is a fundamental way of resolving this problem. With the development of GWAS and transcriptomic microarray technology, we now have powerful research tools to identify SS2 resistance genes.

**Results:**

In this research, we generated an F_2_ generation of SS2 resistant C57BL/6 and SS2 susceptive A/J mice. With the F_2_ generation of these two mice strains and GWAS analysis, we identified 286 significant mouse genome SNPs sites associated with the SS2 resistance trait. Gene expression profiles for C57BL/6 and A/J were analyzed under SS2 infection pressure by microarray. In total, 251 differentially expressed genes were identified between these two mouse strains during SS2 infection. After combining the GWAS and gene expression profile data, we located two genes that were significantly associated with SS2 resistance, which were the UBA domain containing 1 gene (Ubac1) and Epsin 1 gene (Epn 1). GO classification and over-representation analysis revealed nine up-regulated related to immune function, which could potentially be involved in the C57BL/6 SS2 resistance trait.

**Conclusion:**

This is the first study to use both SNP chip and gene express profile chip for SS2 resistance gene identification in mouse, and these results will contribute to swine SS2 resistance breeding.

**Electronic supplementary material:**

The online version of this article (10.1186/s12864-018-5339-9) contains supplementary material, which is available to authorized users.

## Background

*Streptococcus suis* serotype 2 (*S. suis* 2, SS2) is the major pathogen of swine streptococcosis. It has a significant impact on the development of swine industries worldwide, and is an emerging zoonotic agent capable of infecting humans. Among the 33 known serotypes (1–31, 33, 1/2), SS2 has the highest virulence and is the most frequently isolated serotype from animals [1–3]. Although SS2 virulence factors have been extensively studied, few researches focus on the host side; the involvement of specific genes with host susceptibility is poorly understood. Moreover, current major preventative and treatment measures of Swine SS2 infection are vaccination and drugs, which are of limited effectiveness. Along with the development of transgenic technology, disease-resistant breeding provides new opportunities for the prevention and treatment of Swine SS2 infection.

The clinical symptoms of mice infected with SS2, including meningitis, endocarditis, arthritis and septicemia, are similar to those of infected swine [4]. Diverse inbred mouse strains have different susceptibilities to bacterial infection and this animal model has been previously used to identify pathogen resistance genes in the host genome [5–7]. Previous reports identified C57BL/6 and A/J mouse strains as having different susceptibilities to SS2 infection; the C57BL/6 mouse was more resistant to SS2 while the A/J mouse had higher mortality from SS2 infection [8]. Thus, the crossed population of these two mouse strains is suited to locate SS2 resistance genes within the mouse genome.

With the rapid development of bioengineering and bioinformatics, gene chips have been widely used in genomic studies. Genome-wide association study (GWAS) is an effective method that leverages statistical tools to examine the whole genome-wide genetic association between genetic variants and observable traits through microsatellite markers or Single Nucleotide Polymorphisms (SNPs) [9]. This approach has been successfully applied to identify bacterial pathogen susceptibility genes [6, 7]. For the most part, GWAS results only locate the quantitative trait loci (QTL), and further experiments are often needed to further refine the exact gene location [6, 10, 11]. Gene expression profiling can be used to perform high-throughput examination of gene functions and expression conditions, as well as analyzing the regulatory function of single or multiple genes in gene regulatory networks. Combination of these two powerful tools will greatly benefit the identification of SS2 resistance genes from the host genome.

In this study, we aimed to identify genes associated with susceptibility to SS2 through SNP genotyping chips and gene transcription chips. Firstly, we determined the differential susceptibility between A/J mice and C57BL/6 mice and generated F_2_ progeny. Then we obtained genomic DNA (Deoxyribonucleic acid) samples from F_2_ progeny and used SNP genotyping chips to locate significant SNPs with the samples. Next, we collected blood samples from A/J mice and C57BL/6 mice at different hours after SS2 challenge and used the blood samples to identify differentially expressed genes through gene expression profiling. Finally, to refine the association of genes with SS2 susceptibility, we integrated results of significant SNPs and DEGs and determined candidate genes, which were identified as potential SS2 resistance breeding genes.

## Results

### Differential SS2 susceptibility between a/J and C57BL/6

**Fig. 1:**
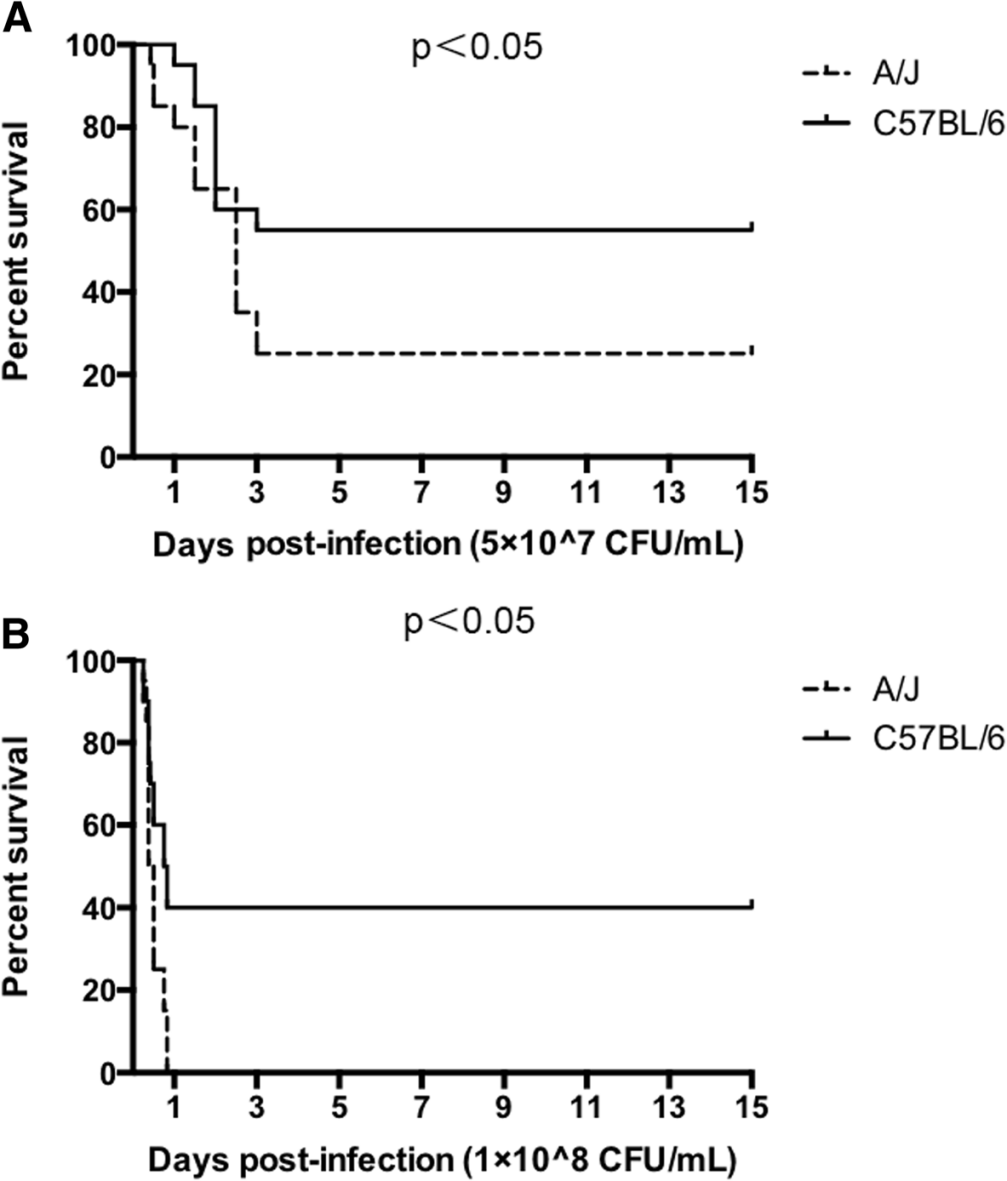
Survival curves of A/J and C57BL/6 mice after infection with *S. suis* serotype 2. **a** Mice were injected i.p. with 1 mL 5 × 10^7^ CFU/mL SS2. **b** Mice were injected i.p. with 1 mL 5 × 10^8^ CFU/mL. Mortality of each group was recorded daily for 15 days. A/J mice showed significantly higher susceptibility to SS2 compared with C57BL/6 mice. (*P*<0.05 with Log-rank Test)

Relatively susceptible mice displayed clinical signs such as rough hair coat, unusual postures, swollen eyes and inability to stand after injection. Most of the susceptible mice died while a few of them recovered. In general, C57BL/6 mice showed stronger resistance compared with A/J mice (Fig. 1). When the inoculum doses for SS2 infection were 5 × 10^7^ CFU/mL and 1 × 10^8^ CFU/mL, A/J mice showed higher susceptibility to SS2 than C57BL/6 mice. These results also provided the suitable challenge dosage for our further studies, which was 1 × 10^8^ CFU/mL; at this dose, SS2 successfully produced significant susceptibility differences between A/J and C57BL/6 mice. According to these results, there should be some specific differential gene expression between these two strains of mice related to the complex reactions arising from SS2 infection.

### Identification of SS2 resistant and susceptible mice from F_2_ progeny of a/J and C57BL/6

**Fig. 2:**
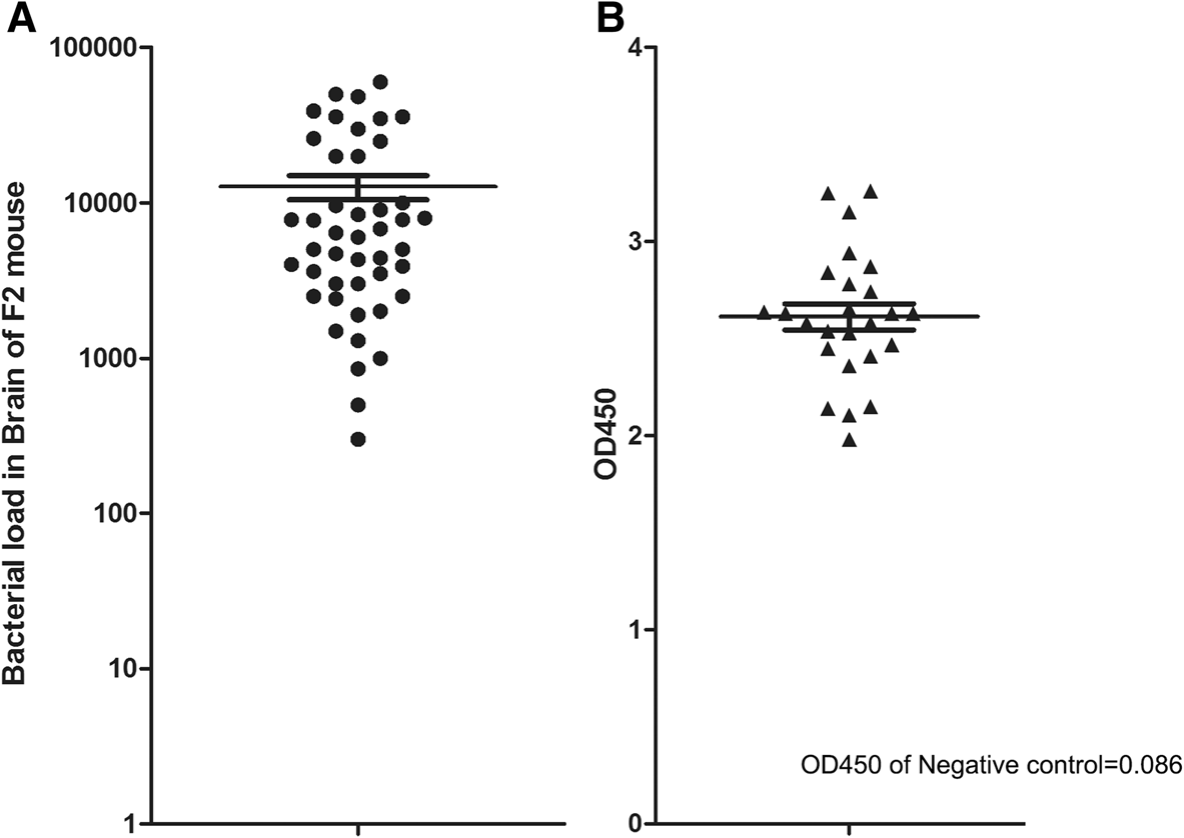
Bacterial loads and antibody titers of F_2_ progeny mice. **a** SS2 can be isolated from brains of all susceptible F_2_ mice. Bacterial loads are calculated as CFU from 0.05 g brain tissue. **b** ELISA results show that all sera of SS2 resistant F_2_ mice are SS2 antibody positive. Negative control sera were isolated from uninfected F_2_ mice. ELISA results were read at an optical density (OD) 450 nm

The F_2_ progeny of A/J and C57BL/6 were challenged with SS2 via intraperitoneal (i.p.) injection; mice that died within 3 days were defined as susceptible while those that lived more than 15 days were defined as resistant. Bacterial loads in brains of susceptible mice were determined to ensure that all mice died due to SS2 infection (Fig. 2a). Three days are too short for the these mice to generate detectible antibody titer, so we did not show their antibody titer data. Meanwhile, the antibody titers of resistant mice were determined through indirect ELISA to ensure that all surviving mice were successfully challenged with SS2 (Fig. 2b). Otherwise, the bacterial load in brain of these mice were counted, no SS2 was detected. Thirty-four resistant mice (18 female and 16 male) and 45 susceptible mice (22 females and 23 males) were identified and used for further GWAS based on SNP chips.

### GWAS of SS2 resistance genes on chromosomes of F_2_ progeny through significant SNPs

**Fig. 3:**
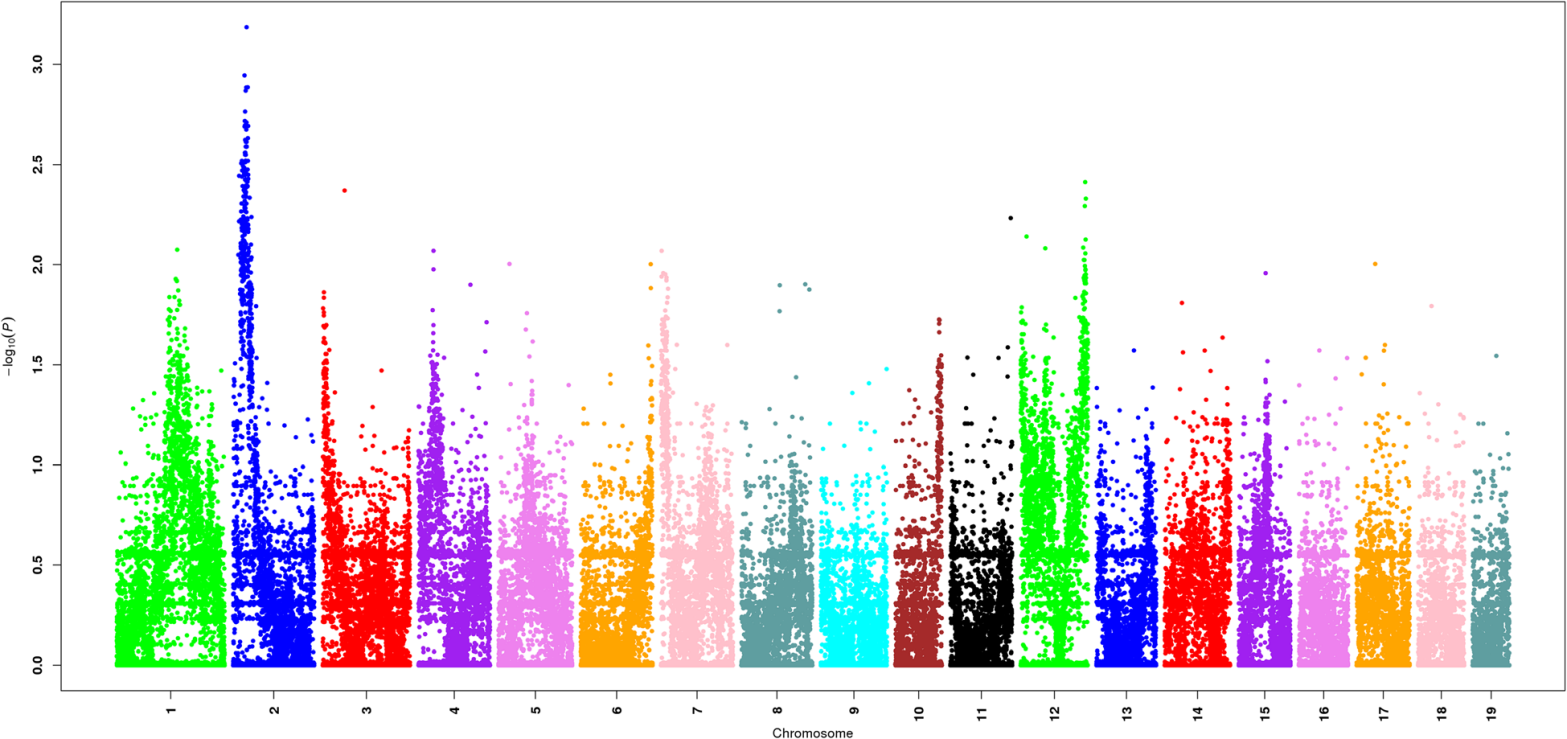
Manhattan plot of F_2_ susceptible and F_2_ resistant samples. Significant association signals were observed on the qB, qC1.1 cytobands of chromosome 2. Compared to other chromosomes, SNPs on qA1, qE3 cytobands of chromosome 7 and qF2 cytobands of chromosome 12 are also significant. Thus, these SNPs may be related to mouse SS2 susceptibility

Thirty-four resistant F_2_ mice and 45 susceptive F_2_ progeny mice were included in this study. Genomic DNA samples were extracted from livers and passed quality control. According to the population structure (Additional file 1: Figure S3), the genetic backgrounds of F_2_ mice were consistent; this showed that the comparison between resistant samples and susceptible samples was feasible. In Q-Q plot (Additional file 1: Figure S4), real values were in accordance with predicted values; thus, the results scanned from SNP genotyping chips were reliable. We applied quality control measures to remove invalid SNP sites with call rates of less than 95% or minor allele frequencies (MAFs) values of zero. Finally, 150,239 SNPs were qualified for association analysis. According to the Bonferroni correction method, the corrected *P*-value as threshold for significant SNPs was expected to be 10^–2.5^. SNPs with *P*-values lower than 10^–2.5^ may be associated with resistance to SS2, which is shown in the Manhattan plot (Fig. 3). SNPs in some of the mouse chromosomes were identified as associated with SS2 resistance (Table 1). Significantly gathered association signals are present on the qB and qC1.1 cytobands of chromosome 2; in addition, SNPs on the qF2, qF1 and qA1.1 cytobands of chromosome 12 are also significant compared to those on other chromosomes (Additional file 2: Table S1); although these are likely linked to SS2 resistance genes of F_2_ mice, further research will be required for confirmation.

**Table 1:** Distribution of SNPs in mouse chromosomes

Chromosome	1	2	3	4	5	6	7	8	12	14	17	All
Significant SNPs	1	279	1	3	1	1	1	2	5	1	1	296

### Different expression genes between C57BL/6 and a/J following infection with SS2

**Fig. 4:**
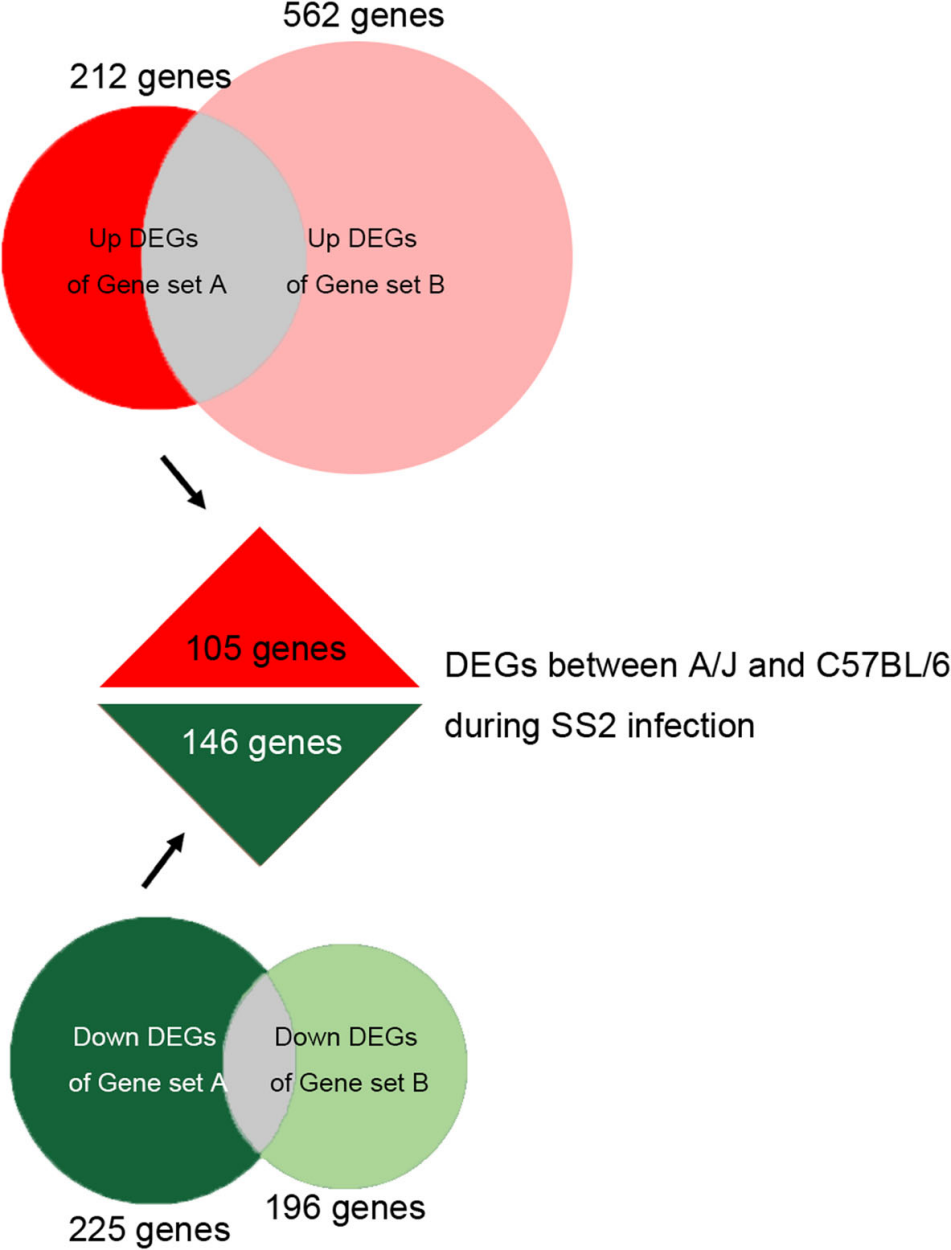
Schematic of different expression genes between C57BL/6 and A/J following infection with SS2. There are 105 up-regulated and 146 down-regulated genes in C57BL/6 relative to A/J mice

**Fig. 5:**
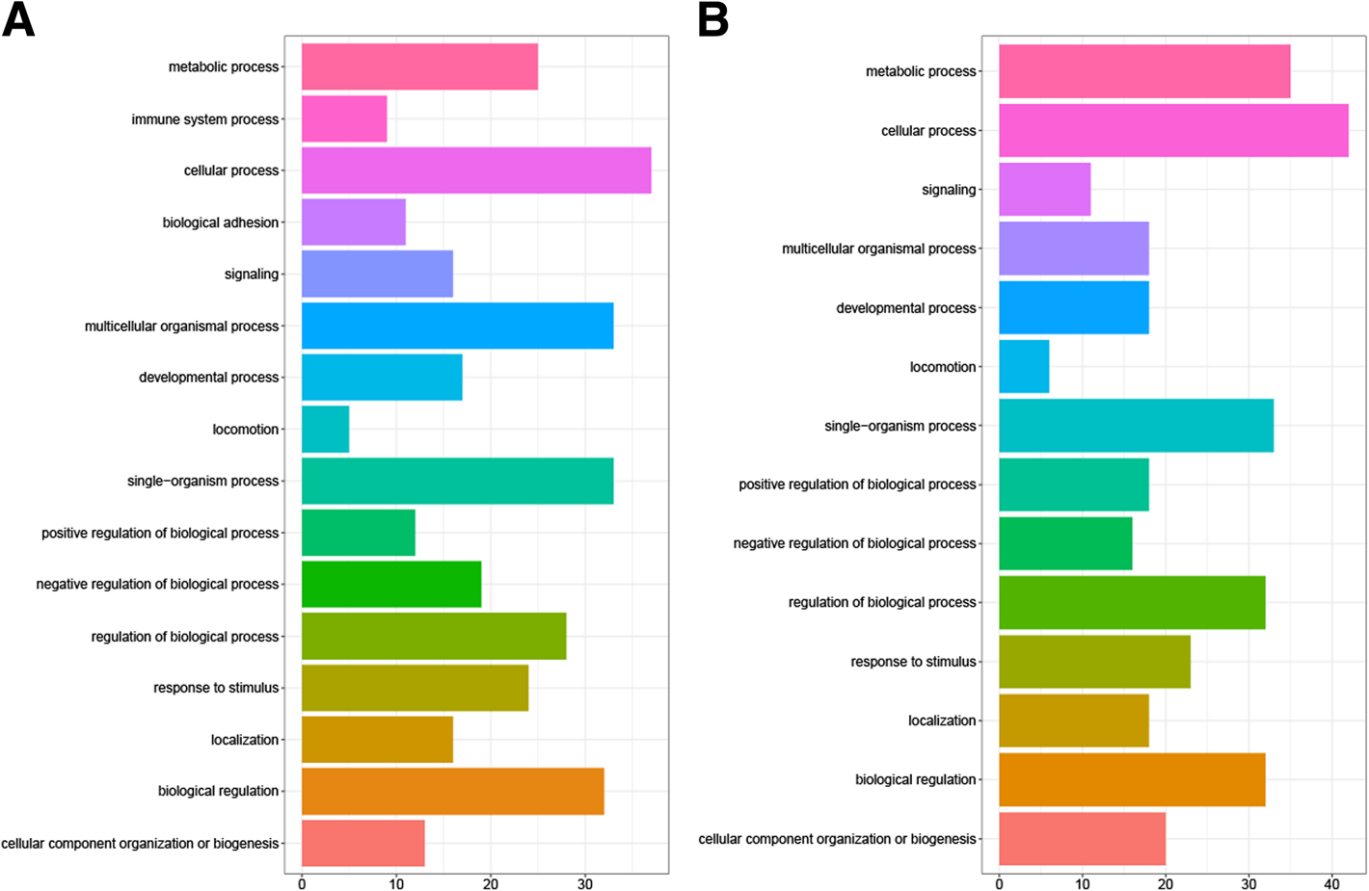
GO classification (biological process) of 56 up-regulated genes (**a**) and 73 down-regulated genes (**b**) with known Entrez ID. The x-axis indicates the number of genes for each GO term

**Fig. 6:**
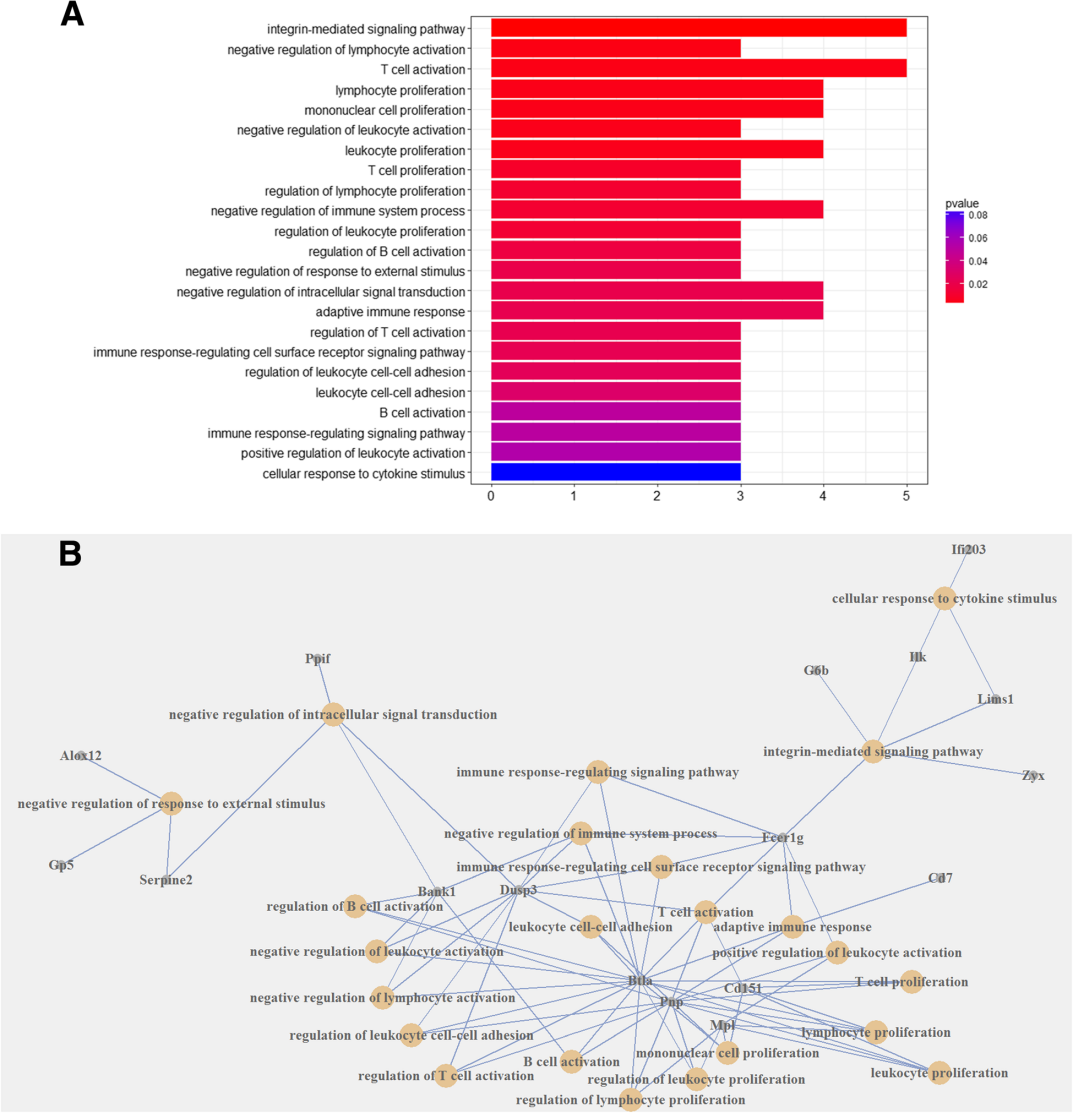
GO over-representation analysis. **a** Up-regulated immunity related GO terms. Larger than expected numbers of up-regulated genes associated with the GO term biological processes are indicated by *P*-values. **b** Connections for the immunity related GO terms and their genes

The differentially expressed genes (DEGs) were analyzed in four independent groups as shown in Table 2. The numbers of DEGs in each group are shown in Table 3. We identified DEGs in common between groups 1, 2 and 3 and assigned these to Gene set A (Additional file 3: Table S2). The DEGs within group 4 were defined as Gene set B (Additional file 4: Table S3). The genes belonging to Gene set A but undetected in Gene set B were considered as DEGs between A/J and C57BL/6 during SS2 infection (Fig. 4). All these 105 up-regulated genes and 146 down-regulated genes are listed in Additional file 5: Table S4. The 56 up-regulated genes and 73 down-regulated genes with known Entrez IDs were used for biological process (BP) GO (Gene Ontology) classification and GO over-representation analysis. The GO classification results are shown in Fig. 5; there are nine up-regulated genes in C57BL/6 belonging to the immune system process, but no down-regulated genes were identified in this category. Detailed GO classification results are listed in Additional file 6: Table S5. Further analyses of GO over-representation focused on the up-regulated immune related genes. In Fig. 6a, a larger than expected number of up-regulated genes within a GO term biological process is indicated by the *P*-value. Significant over-representation of up-regulated genes included biological processes of lymphocyte activation and proliferation, immune response, cytokine secretory and other immunity. Details of the GO over-representation results are shown in Additional file 7: Table S6. The connections of immunity related genes relative to their GO terms are shown in Fig. 6b.

**Table 2:** Groups of DEGs analysis

Group By	Groups	Description
Infected A/J mice & infected C57BL/BJ mice	1	Infected A/J mice at 2 h after infection vs. infected C57BL/6 mice at 2 h after infection.
	2	Infected A/J mice at 4 h after infection vs. infected C57BL/6 mice at 4 h after infection.
	3	Infected A/J mice at 8 h after infection vs. infected C57BL/6 mice at 8 h after infection.
Uninfected A/J mice & uninfected C57BL/B mice	4	Uninfected A/J mice vs. uninfected C57BL/6 mice.

**Table 3:** The number of DEGs in each group of Table 3

Groups	1	2	3	4
Number of all DEGs	751	1552	1389	758
Number of up-regulated DEGs	392	723	533	562
Number of down-regulated DEGs	359	829	856	196

### Candidate genes related to resistance to SS2

**Fig. 7:**
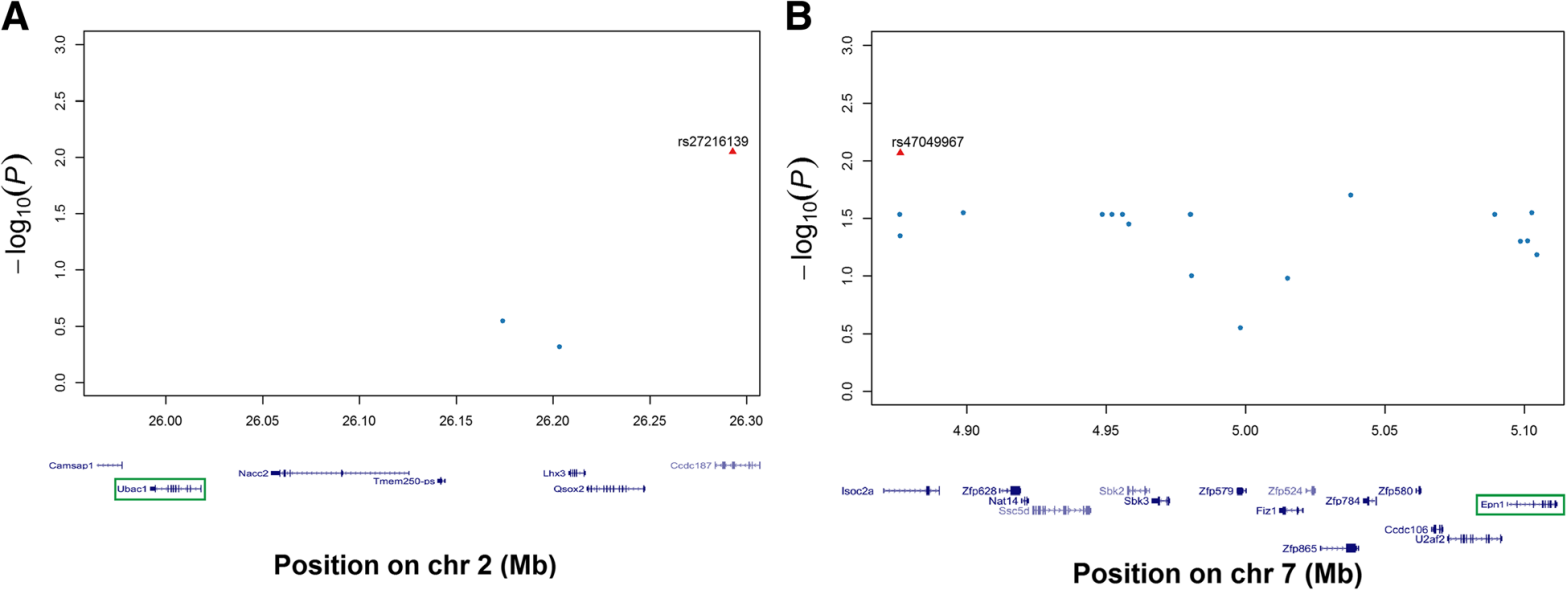
Enlarged Manhattan plots of interest SNP and related DEGs. Enlarged Manhattan plots demonstrate positions of significant SNPs and DEGs alongside cytobands. The red triangle indicates the significant SNP. X-axis shows the scale of chromosome. Strings below X-axis denote genes and their locations. Genes in the green fragment are SNP related DEGs

To locate the exact genes associated with resistance and susceptibility to SS2 infection in mouse, results from SNP genotyping and gene expression profiling were combined and calculated collectively. The significant SNPs (rs27216139 and rs47049967) and DEGs distributed in chr2 (chromosome) and chr7 (Additional file 8: Table S7) have *P*-values lower than 0.01, which indicates significant correlations to the mouse SS2 resistant trait. The 500 kb segments above and below significant SNPs were set as a high correlation range to the SS2 susceptibility phenotype. In the 500 kb upstream range of SNP rs27216139, the DEG Ubac1 (ubiquitin associated domain containing 1) was identified (Fig. 7a). In addition, the DEG Epn1 (Epsin1) was identified in the 500 kb downstream range of SNP rs47049967 (Fig. 7b). Enlarged Manhattan plots were drawn to describe relationships among significant SNPs and DEGs. These two genes are likely involved in the A/J mouse SS2 susceptibility trait, and thus, these should be followed up with functional studies.

## Discussion

SS2 is one of the most serious bacterial pathogens in swine; as an emerging zoonotic infection, it frequently infects humans working closely with swine. In China and Europe, it is often considered as the most virulent and prevalent pathogen involved in swine infections [12]. With the development of genetic modification technology, it has become possible to protect domestic animals from pathogen infection through modifying their genomes [13, 14]. For genomic modification, we have to locate the target genes determinate for SS2 resistance traits. GWAS has been widely used in identifying trait related genes in plants and animals, but at best, it can only narrow the location to a chromosomal region rather than an exact gene. In this study, we employed the transcriptome results to amend GWAS data for a more accurate identification of SS2 resistant gene locations in a mouse model.

Most domestic animals, such as pigs, are expensive and hard to generate a large group to meet the requirement of GWAS analysis. As a reliable animal model, mice have been frequently used in identification of resistant genes to bacterial infection [5, 7, 11]. In this study, we used the SS2 susceptible A/J mouse and SS2 resistant C57BL/6 mouse in the GWAS and transcriptome analysis. Our study showed a significant difference in SS2 resistance between C57BL/6 and A/J mice with an infectious dose of 1 × 10^8^ CFU/mL; these results successfully determined their respective SS2 susceptibility and resistance in agreement with the conclusion of M. Gottschalk [8], and thereby sets a solid foundation for screening of SS2 resistance genes. Although the A/J mouse is known as a C5 complement defective strain [15], there is no conclusive evidence indicating that this deficiency is responsible for pathogen susceptibility of A/J [16]. Moreover, M. Gottschalk also demonstrated that complement is not essential for SS2 susceptibility of A/J [8], which indicated that other genes must be responsible for A/J SS2 susceptibility. We also found that the SS2 resistance trait in C57BL/6 or A/J had no connection with genders (data not show), so in furthering these studies, we only focused on the euchromosome.

In order to identify related genes that are resistant to Anti-Swine SS2 infection, the analysis was conducted by leveraging the results of SNP chip and the gene express profile results. We used the 500 kb segments above and below significant SNPs as extension interval, and screened differentially expressed genes within that range. We further narrowed down the range of candidate genes that are resistant to Swine Anti-Swine SS2 infection according to enlarged Manhattan plots and related literature, including the ubiquitin-associated domain containing 1 gene (Ubac1) and Epsin1 gene (Epn1). These two genes were both down-regulated in the SS2 resistant mouse. Ubac1 is also known as KPC2 (Kip1 ubiquitylation-promoting complex 2), which acts together with KPC1 to form the ubiquitin ligase KPC and regulate degradation of the cyclin-dependent kinase inhibitor p27 [17]. p27 plays a pivotal role in the control of cell proliferation [18]. A significant lowering of B lymphocytes has been shown in a p27 knockdown mouse relative to wild type, which suggests that reduced p27 could block the differentiation of from HSCs to B lymphocytes [19]. Mice with lower Ubac1 expression levels will have higher p27 concentrations, and thereby increase the rate of B lymphocyte differentiation. B lymphocytes are a key element in humoral immunity and are crucial for SS2 resistance; however, it is difficult to determine if lower Ubac 1 expression levels are best, and further study is needed to determine the appropriate level. Epsin 1 is involves in the formation of clathrin lattice formation during clathrin-mediated endocytosis (CME) [20], which is a well-characterized process for internalization of molecules or small particles (< 200 nm) at the plasma membrane [21]. It was reported that clathrin-mediated endocytosis is responsible for the characteristic “attaching and effacing” (A/E) lesions on enterocytes during enteropathogenic *Escherichia coli* (EPEC) infection; in addition to clathrin, Epsin1 also colocalized at EPEC attaching sites, and this endocytic component is a crucial factor in the disease process of extracellular pathogens [22]. In our research, the SS2 resistant mouse has lower Epsin 1 expression level, which may protect the host cells from SS2 invasion by decreasing this endocytic effect.

In addition to the combined SNP and expression analysis results, these two experiment separately provided important information for SS2 resistance genes. The SNP results helped determine some noteworthy physical genomic locations, which may be relevant to mouse SS2 resistance. With the GeneChip® Mouse Transcriptome Assay 1.0 microarray, we identified many DEGs between A/J and C57BL/6 mice under SS2 infection. There were some intrinsic genes differentially expressed between these two mouse strains, which we had discarded by adding a control group in the microarray analysis. This analysis also ensured that all the remaining 251 genes were differentially expressed in all experiment groups. The GO classification analysis revealed nine DEGs related to the immune function, which should be more closely examined in future studies.

## Conclusion

In conclusion, our research combined analyses of SNP chip gene express profile chip data to improve identification accuracy of genes potentially contributing to SS2 resistance in mouse. After obtaining the differential expression profile between SS2 resistant C57BL/6 and SS2 susceptible A/J mouse strain and combining these data with the SNP results, Ubac 1 and Epn 1 gene were identified as genes related to SS2 resistance. GO classification analysis results of up-regulated genes in C57BL/6 showed there were nine immune related genes, which may play potential roles in SS2 resistance. Further studies of the effects of these genes in imparting SS2 resistance are necessary. This research is the first to take the advantages of both SNP and gene expression profile chips to identify SS2 resistance genes identification in mouse; these results will provide beneficial reference material for genetic variety breeding of SS2 resistance animals.

## Methods

### Ethics statement

Animal experiments were performed under the ethical approval granted by the Nanjing Agricultural University Veterinary College. The Science and Technology Agency of Jiangsu Province approved the protocol. The approval ID is SYXK (SU) 2010–0005. All efforts were made to minimize animals’ suffering. All mice that need to be sacrificed were treated with isoflurane, USP for anesthesia before cervical dislocation.

### Mouse strains and F_2_ progeny

A/J and C57BL/6 mice were purchased from Model Animal Research Center of Nanjing University (Nanjing, China). A/J male mice were mated with C57BL/6 female mice to generate an F_1_ population. For generation of F_2_ progeny, F_1_ male mice were mated with inbred F_1_ female mice (Additional file 1: Figure S1). All the mice were raised to 8–10 weeks old in the Laboratory Animal Center of Nanjing Agricultural University (Nanjing, China), and were allowed to acclimate for more than 7 days with free access to rodent chow and water before experiments.

### Preparation of bacterial strain and experimental infections

*S. suis* serotype 2 strain ZY05719 was used in susceptibility studies and experimental infection. For preparation of ZY05719 for injection, bacteria were cultured overnight on Todd Hewitt broth (THB) (Oxoid Ltd., USA) agar plates at 37 °C; single colonies were inoculated into 5 ml of THB, which was incubated for 8 h at 37 °C with agitation. Cultures were transferred into THB at a 1:100 dilution, which was incubated for 16 h to stationary phase under the same conditions. Bacteria were harvested by centrifugation, washed twice in phosphate buffered saline (PBS) (pH 7.3) and re-suspended in THB. The optical density at wavelength 600 nm (OD_600_) of the suspension was measured with a spectrophotometer, and the suspension was diluted to apposite concentrations according to experimental requirements.

Of the 284 mice included in the study, 60 A/J and 60 C57BL/6 were for susceptibility testing, 140 F_2_ mice were for SNP genotyping chip, and 12 A/J and 12 C57BL/6 were for gene expression profiling; all mice were injected with 1 mL fresh ZY05719 suspension at 1 × 10^8^ CFU/mL via an intraperitoneal (i.p.) route.

### Determination of susceptible mice and bacterial loads in brains

The inoculum for experimental infection was adjusted to concentrations of 5 × 10^7^ CFU/mL, 1 × 10^8^ CFU/mL and 5 × 10^8^ CFU/mL. A total of 120 mice were randomly divided into 3 experimental groups, each of which concluded 10 female A/J mice, 10 male A/J mice, 10 female C57BL/6 mice and 10 male C57BL/6 mice. Each group was injected i.p. with the above doses (1 mL per mouse), respectively. Mortality and clinical signs of Streptococcosis disease were closely observed and recorded. Mice surviving for fewer than 3 days after infection were defined as susceptible.

Brains of susceptible mice were obtained immediately and aseptically after their death. The organs were then trimmed to 0.05 g, placed in 500 μL PBS and homogenized through a vortex. Serial dilutions (ten-fold) of homogenates in PBS were coated on THB agar plates and incubated overnight at 37 °C; all samples were plated in triplicate. Colonies on each plate were counted and calculated.

### Determination of resistant mice and antibody titer detection with ELISA

Mice surviving for more than 15 days after SS2 infection were defined as resistant. Blood samples of resistant mice were harvested aseptically by extraction by retro-orbital plexuses. Serum samples were collected from corresponding blood samples and used for enzyme-linked immunosorbent assay (ELISA). The 96-well plates were coated with muramidase-released protein (MRP) and incubated overnight at 4 °C. The plates were washed three times with phosphate-buffered saline containing 1% Tween 20 (PBST), and blocked with PBST containing 5% non-fat dried milk or bovine serum albumin (BSA). After 2 h incubation at 37 °C, the plates were washed another three times with PBST. Two fold serial dilutions of serum samples in PBST were added to the plates and incubated for 2 h at 37 °C. Plates were washed 3x with PBST, optimally diluted (1:10000) Staphylococcal protein A-Horse Radish Peroxidase (SPA-HRP) was added, and the plates were incubated at 37 °C for 1 h. The plates were then subjected to the last three PBST washes, and 3,3′,5,5’-Tetramethylbenzidine (TMB) was added to each well. The reactions were stopped by adding 2 M H_2_SO_4_, and OD_450_ absorbance values were read using a microplate reader.

### Preparation of genomic DNA and SNP genotying chip assay

The SS2 inoculum for experimental infection was adjusted to a concentration of 1 × 10^8^ CFU/mL. Seventy male and seventy female F_2_ mice were injected i.p. with a 1 mL inoculum per mouse. F_2_ mice surviving for fewer than 3 days following infection were regarded as susceptible (22 males and 22 females), while those surviving for more than 15 days were regarded as resistant (23 males and 21 females). Blood samples were collected aseptically from susceptible and resistant F_2_ mice. Genomic DNA was extracted from blood using TIANamp Genomic DNA Kit, and then amplified and hybridized to an Affymetrix Mouse Diversity Genotyping Array (Affymetrix) according to the manufacturer’s instructions (Affymetrix Inc.). The overall hybridization quality was estimated by the call rate index obtained from Genotyping Console Software (GTC 4.0, birdseed algorithm with default parameter settings). Mixed Linear Model (MLM) of GCTA software was used for GWAS. PLINK 1.09 was used to analyze the four Principal Component Analysis (PCA) values C1-C4, and these were used to correct the population structure with MLM. The Bonferroni method was modified via Linkage Disequilibrium (LD) and used to correct *P*-values of SNPs. 500 SNPs were set as a window, among which, 50 SNPs were set as a walking and the threshold of r^2^ was set as 0.2; thus, the SNP significance threshold was set at a bonferroni corrected P-value of less than 5%. SNPs were compared between samples derived from sensitive and resistant mice. Manhattan plots and quantile-quantile plots (Q-Q plot) were drawn to explore significant SNPs, which might be related to SS2 susceptibility.

### Preparation of total RNA and transcriptome analysis

The inoculum for experimental infection was adjusted to a titer of 1 × 10^8^ CFU/mL. Twenty-four 8–10 week old A/J and C57BL/6 mice were randomly divided into four groups, each of which contained 3 A/J and 3 C57BL/6 mice. Mice in the three experimental groups were injected with the inoculum (1 mL per mouse) via i.p., and their blood samples were collected aseptically at 2, 4 and 8 h after injection respectively. Mice in the fourth control group had blood samples aseptically taken at the same intervals following injection with 1 mL of THB culture. Total RNA was extracted from blood samples using TRIpure LS Reagent, and tested for quality control. 1st cycle 1st strand cDNA, 1st cycle 2nd strand cDNA and 2nd cycle 1st strand cDNA were synthesized successively. The latter synthesis product was then fragmented and labeled. GeneChip® Mouse Transcriptome Assay 1.0 (Affymetrix) was used for the microarray assay. Affymetrix GeneChip Command Console® (AGCC) software was used to export. DAT fluorescence images as. JPG and. CEL files. The. CEL file information was corrected with Robust Multi-chip Average (RMA), and the probe signals were integrated as probe set signals using Expression Console (EC) software. The .chp files obtained through EC were analyzed using Transcriptome Analysis Console (TAC) software to conduct transcriptome analysis. The standards to identify differentially expressed genes (DEG) were fold change (FC) ≥ |2| with a *P*-value ≤0.05.

To identify DEGs related to SS2 resistance expressed between A/J and C57BL/6 mice, comparison schemes of differentially expressed genes between experimental groups were created as 4 groups, Infected A/J mice at 2 h (1), 4 h (2), 8 h (3) after infection vs. infected C57BL/6 mice at 2 h (1), 4 h (2), 8 h (3) after infection respectively and uninfected A/J mice vs. uninfected C57BL/6 mice (4). After preliminary analysis of DEGs between different groups, DEGs specific to mouse strains were obtained through a data processing plan. We identified DEGs in common between groups 1, 2 and 3 and assigned these to Gene set A. The DEGs within group 4 were defined as Gene set B. The genes belonging to Gene set A but undetected in Gene set B were considered as DEGs between A/J and C57BL/6 during SS2 infection. The R package “clusterProfiler” was employed for bioinformatics analysis of these DEGs [23].

### Data integration

To further screen for genes, which could account for resistance to *S. suis* infection, results of SNP genotyping chips and gene express profile chips were integrated (Additional file 1: Figure S2). The SNP significance threshold was set to a *P*-value of 0.01. The 500 kb segments up- and downstream of significant SNPs were set as extension intervals. DEGs located within the intervals were selected and named as candidate genes. Enlarged Manhattan plots were drawn to demonstrate the relationship between the *P*-values of SNPs and the position of DEGs along with chromosomes. Genes of interest were chosen from candidate genes according to enlarged Manhattan plots and related literature.

## Additional files

Additional file 1: **Figure S1**. Breeding scheme used to generate F_2_ mice. Since the fertility of C57BL/6 female mice is higher than A/J female mice, we chose C57BL/6 female mice and A/J male mice as parents to generate F_1_ progeny. To avoid inbreeding, F_1_ male mice were mated with F_1_ female mice from a different group to generate the F_2_ mice. **Figure S2**. Overall strategy for identifying genes associated with resistance to *S. suis* in swine. Flow chart of the strategy for identifying *S. suis* resistant genes in swine through gene expression profiling and SNP genotyping chip. **Figure S3**. Population structure analyzed from PCA values. Principal component values (C1, C2 and C3) were obtained through PLINK1.09 and used to correct population structure. Each point stands for a sample in the plots. Although points are concentrated in (A) and (B), and scattered in (C), none formed into several clusters in the three plots, showing the genetic background was consistent in F_2_ mice. **Figure S4**. Q-Q plot of F_2_ susceptible samples and F_2_ resistant samples. The plot showed deviation between expected *P*-values and observed *P*-values. Each point stands for a SNP. Points on the diagonal of coordinate were considered as non-influential to SS2 susceptibility, while points deviating from the diagonal might be associated with the trait. (PDF 594 kb)

Additional file 2: **Table S1**. Details of significant SNPs related to SS2 susceptibility. (XLSX 21 kb)

Additional file 3: **Table S2**. Duplicated DEGs among groups 1, 2 and 3 of Table 3. (XLSX 195 kb)

Additional file 4: **Table S3**. DEGs in group 4 of Table 3. (XLSX 255 kb)

Additional file 5: **Table S4**. DEGs between A/J and C57BL/6 during SS2 infection. (XLSX 69 kb)

Additional file 6: **Table S5**. Details of GO classification. (XLSX 11 kb)

Additional file 7: **Table S6**. Details of GO over-representation. (XLSX 12 kb)

Additional file 8: **Table S7**. SNPs significant to SS2 susceptibility and corresponding DEGs located within their 500 kb high correlation range. (XLSX 10 kb)

## Abbreviations

A/E: Attaching and effacing
BP: Biological process
CFU: Colony-forming unit
Chr2: Chromosome
CME: Clathrin-mediated endocytosis
DEGs: Differentially expressed genes
DNA: Deoxyribonucleic acid
ELISA: Enzyme-linked immunosorbent assay
EPEC: Enteropathogenic *Escherichia coli*
Epn1: Epsin1
GO: Gene Ontology
GWAS: Genome-wide association study
i.p.: Intraperitoneal
KPC2: (Kip1 ubiquitylation-promoting complex 2)
LD: Linkage Disequilibrium
MAFs: Minor allele frequencies
MRP: Muramidase-released protein
PBS: Phosphate buffered saline
PBST: Phosphate-buffered saline containing 1% Tween 20
PCA: Principal Component Analysis
QTL: Quantitative trait loci
SNPs: Single nucleotide polymorphisms
SPA-HRP: Staphylococcal protein A-Horse Radish Peroxidase
SS2: Streptococcus suis serotype 2
THB: Todd Hewitt broth
TMB: 3,3′,5,5’-Tetramethylbenzidine
Ubac1: Ubiquitin associated domain containing 1
