# Supplementary material for: Screening of Streptococcus Suis serotype 2 resistance genes with GWAS and transcriptomic microarray analysis
Source: BMC Genomics. 2018 Dec 12;19:907. doi: 10.1186/s12864-018-5339-9 (PMC6292034; doi:10.1186/s12864-018-5339-9)
Supplement: Supplementary file 1 — Figure S1. Breeding scheme used to generate F2 mice. Since the fertility of C57BL/6 female mice is higher than A/J female mice, we chose C57BL/6 female mice and A/J male mice as parents to generate F1 progeny. To avoid inbreeding, F1 male mice were mated with F1 female mice from a different group to generate the F2 mice. Figure S2. Overall strategy for identifying genes associated with resistance to S. suis in swine. Flow chart of the strategy for identifying S. suis resistant genes in swine through gene expression profiling and SNP genotyping chip. Figure S3. Population structure analyzed from PCA values. Principal component values (C1, C2 and C3) were obtained through PLINK1.09 and used to correct population structure. Each point stands for a sample in the plots. Although points are concentrated in (A) and (B), and scattered in (C), none formed into several clusters in the three plots, showing the genetic background was consistent in F2 mice. Figure S4. Q-Q plot of F2 susceptible samples and F2 resistant samples. The plot showed deviation between expected P-values and observed P-values. Each point stands for a SNP. Points on the diagonal of coordinate were considered as non-influential to SS2 susceptibility, while points deviating from the diagonal might be associated with the trait. (PDF 594 kb) [file 12864_2018_5339_MOESM1_ESM.pdf]

Screening of *Streptococcus Suis* serotype 2 resistance genes with SNP and transcriptomic microarray

Zhe Ma[abc], Haodan Zhu[d], Yiqi Su[ab], Yu Meng[ab], Huixing Lin[abc], Kongwang He[d], Hongjie Fan[abc*]

a. MOE Joint International Research Laboratory of Animal Health and Food Safety, College of Veterinary Medicine, Nanjing Agricultural University, Nanjing 210095, China

b. Ministry of Agriculture Key Laboratory of Animal Bacteriology, Nanjing 210095, China

c. Jiangsu Co-innovation Center for Prevention and Control of Important Animal Infectious Diseases and Zoonoses, Yangzhou 225009, China

d. Jiangsu Academy Agricultural Sciences, Nanjing, 210095, China.

* Corresponding author, Email: fhj@njau.edu.cn

Supplementary

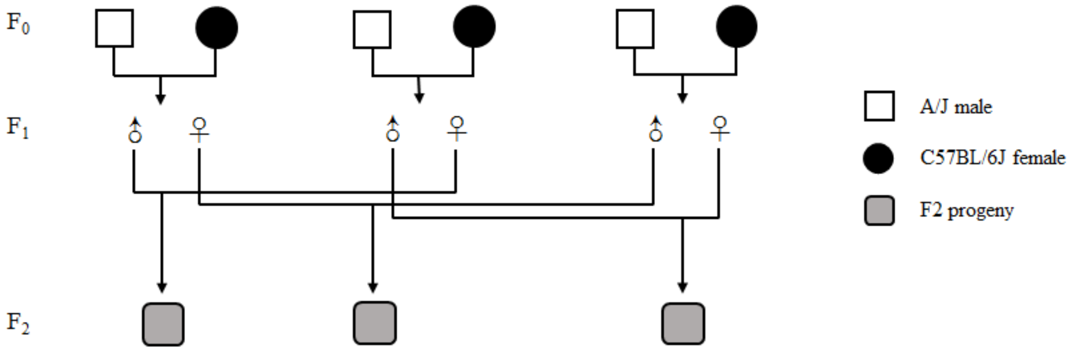

Figure S1. Breeding scheme used to generate F_2 mice. Since the fertility of C57BL/6 female mice is higher than A/J female mice, we chose C57BL/6 female mice and A/J male mice as parents to generate F_1 progeny. To avoid inbreeding, F_1 male mice were mated with F_1 female mice from a different group to generate the F_2 mice.

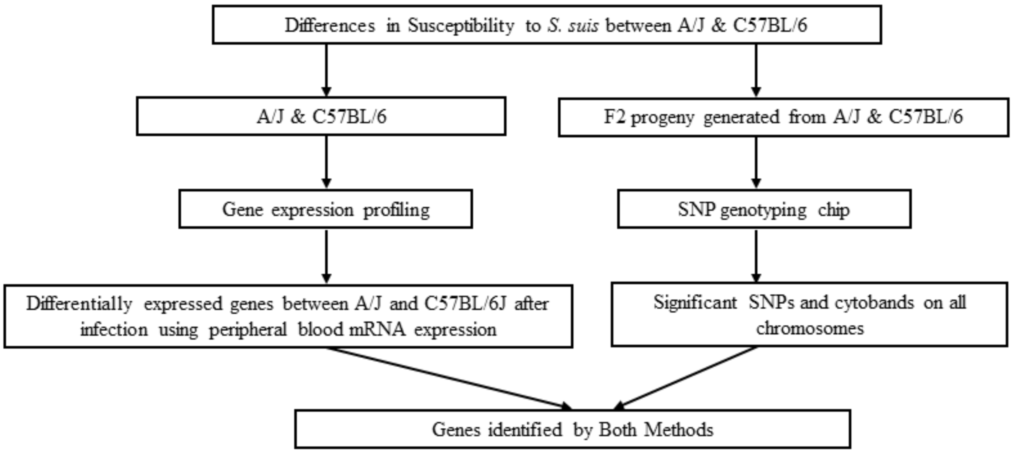

Figure S2. Overall strategy for identifying genes associated with resistance to *S. suis* in swine. Flow chart of the strategy for identifying *S. suis* resistant genes in swine through gene expression profiling and SNP genotyping chip.

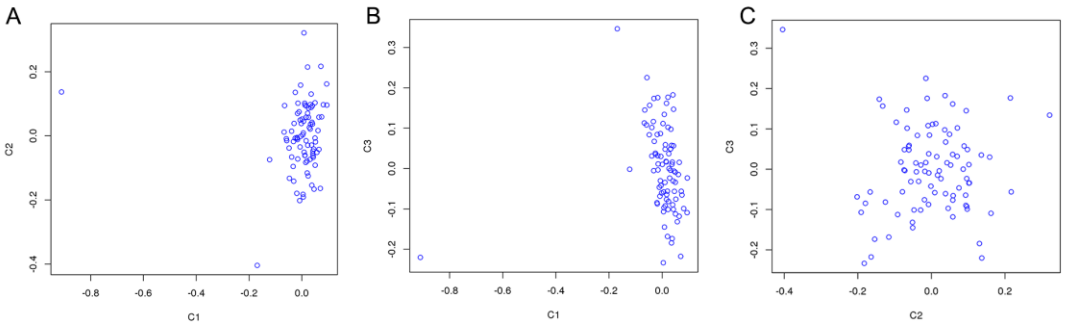

Figure S3. Population structure analyzed from PCA values. Principal component values (C1, C2 and C3) were obtained through PLINK1.09 and used to correct population structure. Each point stands for a sample in the plots. Although points are concentrated in (A) and (B), and scattered in (C), none formed into several clusters in the three plots, showing the genetic background was consistent in F_2 mice.

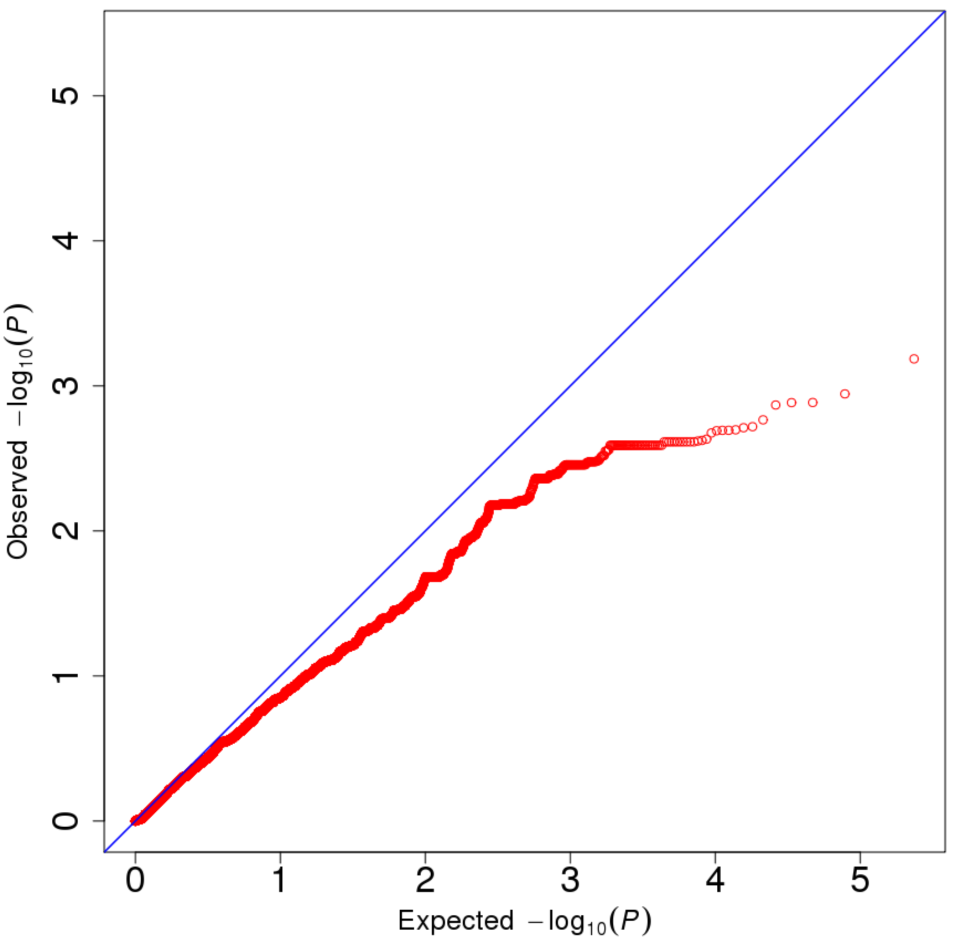

Figure S4. Q-Q plot of F_2 susceptible samples and F_2 resistant samples. The plot showed deviation between expected *P*-values and observed *P*-values. Each point stands for a SNP. Points on the diagonal of coordinate were considered as non-influential to SS2 susceptibility, while points deviating from the diagonal might be associated with the trait.
